# Synchrotron X-Ray Diffraction to Detect Glass or Ice Formation in the Vitrified Bovine Cumulus-Oocyte Complexes and Morulae

**DOI:** 10.1371/journal.pone.0114801

**Published:** 2014-12-23

**Authors:** Muhammad Anzar, Pawel Grochulski, Brennan Bonnet

**Affiliations:** 1 Cryobiology Lab, Canadian Animal Genetic Resource Program, Agriculture and Agri-Food Canada, Saskatoon Research Center, Saskatoon, Saskatchewan, Canada; 2 Department of Veterinary Biomedical Sciences, University of Saskatchewan, Saskatoon, Saskatchewan, Canada; 3 Canadian Light Source, Saskatoon, SK, Canada; Massey University, New Zealand

## Abstract

Vitrification of bovine cumulus-oocyte complexes (COCs) is not as successful as bovine embryos, due to oocyte's complex structure and chilling sensitivity. Synchrotron X-ray diffraction (SXRD), a powerful method to study crystal structure and phase changes, was used to detect the glass or ice formation in water, tissue culture medium (TCM)-199, vitrification solution 2 (VS2), and vitrified bovine COCs and morulae. Data revealed Debye's rings and peaks associated with the hexagonal ice crystals at 3.897, 3.635, 3.427, 2.610, 2.241, 1.912 and 1.878 Å in both water and TCM-199, whereas VS2 showed amorphous (glassy) appearance, at 102K (−171°C). An additional peak of sodium phosphate monobasic hydrate (NaH_2_PO_4_.H_2_O) crystals was observed at 2.064 Å in TCM-199 only. All ice and NaH_2_PO_4_.H_2_O peaks were detected in the non-vitrified (control) and vitrified COCs, except two ice peaks (3.145 and 2.655 Å) were absent in the vitrified COCs. The intensities of majority of ice peaks did not differ between the non-vitrified and vitrified COCs. The non-vitrified bovine morulae in TCM-199 demonstrated all ice- and NaH_2_PO_4_.H_2_O-associated Debye's rings and peaks, found in TCM-199 alone. There was no Debye's ring present in the vitrified morulae. In conclusion, SXRD is a powerful method to confirm the vitrifiability of a solution and to detect the glass or ice formation in vitrified cells and tissues. The vitrified bovine COCs exhibited the hexagonal ice crystals instead of glass formation whereas the bovine morulae underwent a typical vitrification.

## Introduction

Cryopreservation of mammalian oocytes and embryos is important for conservation of female genetics in domestic animals and endangered species [1], [2], and for assisted reproduction in humans [3]. The success of cryopreservation of mammalian oocytes and embryos differs among species, developmental stage and origin [1]. The cryopreservation of bovine oocytes is more difficult than early embryo [4]–[6]. This is mainly due to oocyte's complex structure, i.e. large surface to volume ratio, chilling sensitivity, reduced plasma membrane permeability and low hydraulic conductivity [7]–[9]. Mammalian oocytes and early embryos are commonly cryopreserved by conventional slow freezing or vitrification method. During conventional slow freezing, the ice formation (intra- and extra-cellular), toxicity of cryoprotectant(s), osmotic swelling and shrinkage, and tissue fracture are the common cryoinjuries to mammalian cells [10], [11]. In vitrification, cells are exposed to higher concentrations of permeating cryoprotectants and cooled with ultra-rapid velocity [12]. The vitrified cells/tissues turn into a solid amorphous glass phase bypassing ice formation due to high viscosity of cryoprotectants in cellular compartments [13], [14]. Vitrification has become a popular method of cryopreservation for mammalian oocytes and embryos as it avoids chilling injury and damage due to the intracellular ice formation [15], [16]. Moreover, it is fairly cheap, simple, quick and superior to slow freezing [1], [17]. Vitrification has been successful for mouse oocytes [18], whereas it is still challenging for bovine oocytes [19]. There is no single universal method of vitrification for oocytes and embryos [7]. Like other studies, we also observed poor embryonic development from the vitrified cumulus-oocyte complexes (COCs) as compared with the non-vitrified control COCs [20], [21]. Vitrification causes the lysis of cumulus cells and oocyte, and the misplacement of cortical granules in bovine germinal vesicle (GV) stage COCs [22]. It also causes the disorganization of metaphase plate, condensation of chromosomes and clustering of cortical granules in metaphase II (MII) stage oocytes [23], [24].

The vitrification solutions (VSs) for oocytes and embryo are developed based on empirical or theoretical analyses [25]. The probability of vitrification is directly proportional to viscosity and cooling rate, and inversely proportional to sample volume [26]. The success of vitrification also depends upon warming rate [27]. In vitrification, toxicity of cryoprotectants (CPs) and intracellular ice formation are mainly responsible for the cellular damage [9]. The permeability of plasma membrane to water and CPs varies among cells and tissues [28]. Earlier, we did not observe a significant toxic effect of CPs on bovine COCs [21]. Therefore, it was hypothesized that sufficient quantity of CPs could not reach inside oocytes to manifest their toxic effects and did not increase the intracellular viscosity required for vitrification. Consequently, there could be intracellular ice formation in COCs, following vitrification, which damaged the organelles resulting in poor oocyte maturation, fertilization and embryo development. There is no published report so far to confirm the glass or ice formation in mammalian COCs, embryos or other tissues, upon vitrification.

X-ray diffraction (XRD) is a fast and powerful non-invasive method for phase analysis, i.e. type and quantities of phases in sample, and crystal structure, size and stress [29]. It is used for the quantification of degree of crystallinity. XRD is a result of scattering of X-ray wave from atoms' electrons and is based on “Bragg's law”:
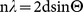
where n (integer) is the “order” of reflection, λ is the wavelength of incident X-ray beam, d is the distance between atomic layers in crystal and θ is the angle of incidence.

In a hexagonal system, which is the case of water crystals, an inverse of the interplanar distance, 1/d^2^, is given by:
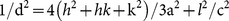
where *h, k* and *l* are the Miller indices which fully describe the set of crystallographic planes, and a and c are cell dimensions.

Synchrotron X-ray radiation is several folds stronger than conventional X-ray which is less sensitive due to the low flux of X-ray source. Synchrotron X-ray diffraction (SXRD) is widely used in pharmaceutical industry especially under extreme conditions of temperature and pressure. SXRD has been successfully used to detect crystallinity in amorphous pharmaceuticals [30], anomalous behaviour of ice during freezing [31] and phase transitions in frozen system [32]. SXRD and 2-dimenstional (2D) area detector possess highly sensitive and rapid data acquisition capability compared to the conventional X-ray instrument [30]. In SXRD, the data of entire Debye's ring are collected and thus errors in the measurements of net intensities of peaks are minimum [33].

The objectives of this study were to determine the vitrifiability of vitrification solution, and to confirm the glass or ice formation in the vitrified bovine COCs and morulae, using SXRD.

## Materials and Methods

### Chemicals and supplies

Dulbecco's phosphate buffered saline (DPBS), newborn calf serum (CS), tissue culture medium (TCM)-199 and MEM non-essential amino acids were purchased from Invitrogen Inc. (Burlington, ON, Canada). Lutropin-V (luteinizing hormone, LH) and Folltropin-V (follicle stimulating hormone, FSH) were supplied by Bioniche Animal Health Inc. (Belleville, ON, Canada). Unless otherwise stated, all other chemicals and reagents were purchased from Sigma-Aldrich (Oakville, ON, Canada).

### Cryodevices

**Figure 1:**
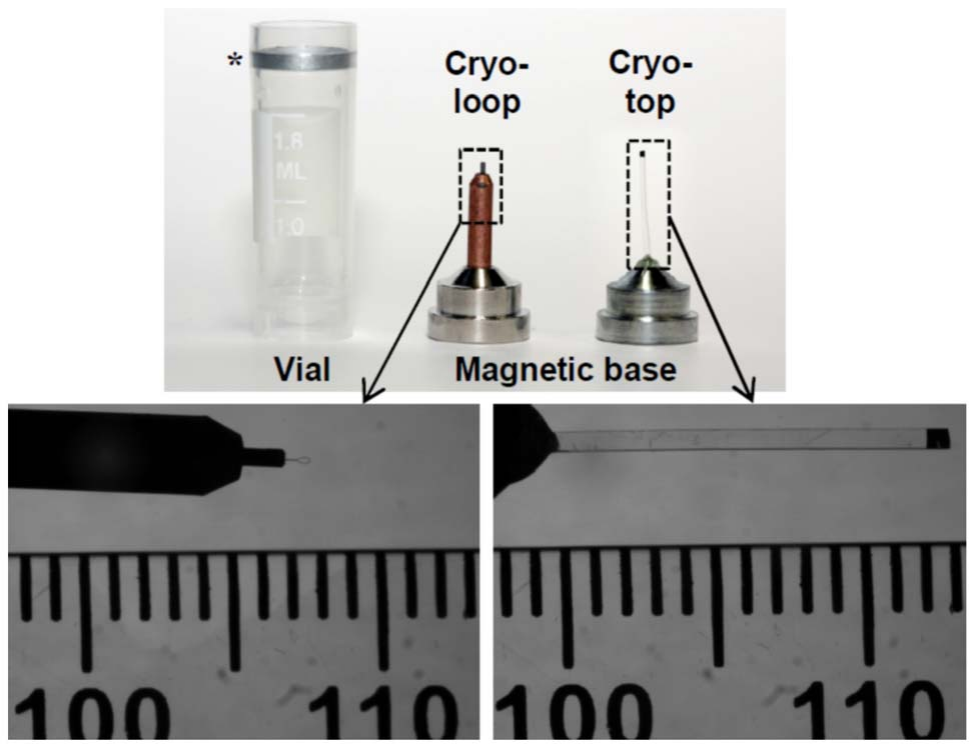
Custom-designed cryoloop and cryotop using CrystalCap Copper Magnetic. Each cap possesses a magnetic base which is fixed on the top of cryovial (*) for storage or goniometer head for SXRD analysis. Cryoloop was used for SXRD of solutions (water, TCM-199 and VS2) and cryotop for bovine COCs and morulae.

Two custom-designed cryodevices, i.e. cryoloop and cryotop, were used in this study (**Fig. 1**). In both cryodevices, CrystalCap Copper Magnetic (Hampton Research, Aliso Viejo, CA) were used. Each cap possesses an alloy base, which magnetically secures the cap in cryovial or on the goniometer head, and a copper pin. A Cryoloop (0.3–0.4 mm in diameter; Hampton Research) was mounted at the end of a copper pin. For a cryotop, the copper pin was replaced with 10–13 mm long propylene strip from original Cryotop (Kitazato Supply Co., Fujinomiya, Japan). Cryoloop was used for the SXRD analysis of water, TCM-199 and vitrification solution 2 (VS2), whereas cryotop was used for the vitrified bovine COCs and morulae.

### COCs collection

Bovine ovaries were procured from a slaughterhouse, operated under strict regulations of Canadian Food Inspection Agency, and transported to laboratory at approximately 25°C. Ovaries were washed in 0.15 M sodium chloride and extra tissues surrounding the ovaries were removed. The follicles (3–8 mm in diameter) were aspirated using an 18-gauge needle attached to 5 ml-syringe containing 1 ml 5% calf serum (CS) in DPBS (vol/vol). The immature (GV stage) COCs were harvested from the pooled follicular fluid under a stereomicroscope at 10× magnification and washed (3x) in 5% CS in DPBS (vol/vol). The COCs with more than three layers of compact cumulus cells and uniform cytoplasm were selected for vitrification or morulae production.

### *In vitro* maturation, fertilization and culture

*In vitro* maturation, fertilization and culture were conducted following the procedures described earlier [20]. The immature COCs were washed (3x) in maturation medium [TCM-199 supplemented with 5% CS, 5 µg/ml LH, 0.5 µg/ml FSH and 0.05 µg/ml gentamicin]. The groups of 20 COCs were placed in each 100 µl-droplets of maturation medium, under mineral oil and incubated at 38.5°C, 5% CO_2_ in air and saturated humidity, for 22 h. For *in vitro* fertilization (IVF), two semen straws from each three fertile bulls were thawed at 37°C for 1 min, pooled and washed through Percoll gradient (45% and 90%) [34]. After washing, sperm were added to Brackett-Oliphant (BO) fertilization medium [35] to final concentration 3×10^6^ cells/ml, and droplets of 100 µl were made under mineral oil. Following IVM, the groups of 20 mature COCs were washed (3x) with 10% bovine serum albumin (BSA) in BO fertilization medium (wt/vol), added to each 100 µl-sperm droplets, and incubated under mineral oil at 38.5°C, 5% CO_2_ in air and saturated humidity. After 18 h coincubation, cumulus cells and sperm attached to COCs were mechanically removed via pipetting. The presumptive zygotes were washed (3x) in *in vitro* culture (IVC) medium CR1aa consisting 5% CS (vol/vol), 2% BME amino acids (vol/vol), 1% MEM nonessential amino acids (vol/vol), 1% L-glutamic acid (vol/vol), 0.3% BSA (wt/vol) and 0.05 µg/ml gentamicin. Zygotes were transferred into 100 µl-IVC droplets under mineral oil and incubated at 38.5°C, 5% CO_2_, 90% N_2_, 5% O_2_ and saturated humidity. On day 6 of culture, morulae were harvested for vitrification.

### Vitrification of COCs and morulae

The COCs (GV stage) and morulae were first equilibrated in 10 ml vitrification solution 1 [VS1; TCM-199 containing 7.5% ethylene glycol (EG; vol/vol), 7.5% dimethyl sulfoxide (DMSO; vol/vol), 20% CS (vol/vol)] in a petri dish (35 mm diameter) for 5 min at 37°C. After equilibration, COCs and morulae were transferred through three 20 µl-microdrops of vitrification solution 2 [VS2; TCM-199 containing 15% EG (vol/vol), 15% DMSO (vol/vol), 20% CS (vol/vol), 17.1% sucrose (wt/vol)] at 37°C for 45–60 sec [16], [36]. The COCs and morulae were loaded quickly on separate cryotops under stereomicroscope, the extra surrounding medium was aspirated as much as possible and immediately plunged in liquid nitrogen [15]. The COCs and morulae in TCM-199 without cryoprotectants were also loaded on cryotops, and plunged in liquid nitrogen as non-vitrified controls for ice formation. The cryotop containing COCs and morulae were placed in the ultra-cooled cryovial and stored in liquid nitrogen until SXRD analysis.

### Synchrotron X-ray diffraction

**Figure 2:**
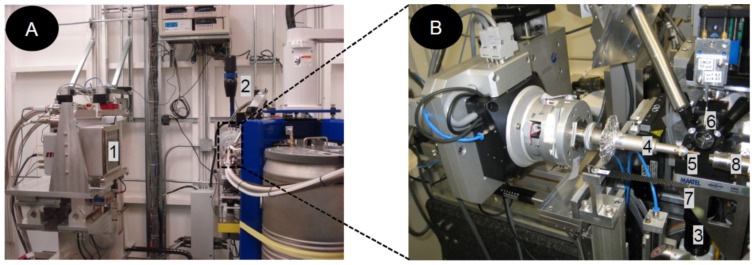
The end-station of beamline 08ID-1 (A) and close-up of sample position area (B). 1) CCD- X-ray detector. 2) Hutch camera for remote monitoring system. 3) Sample camera. 4) Goniometer head. 5) CrystalCap Copper Magnetic mounted on goniometer head. 6) X-ray tube. 7) Beam stop. 8) Liquid nitrogen supply from CryoJet.

**Figure 3:**
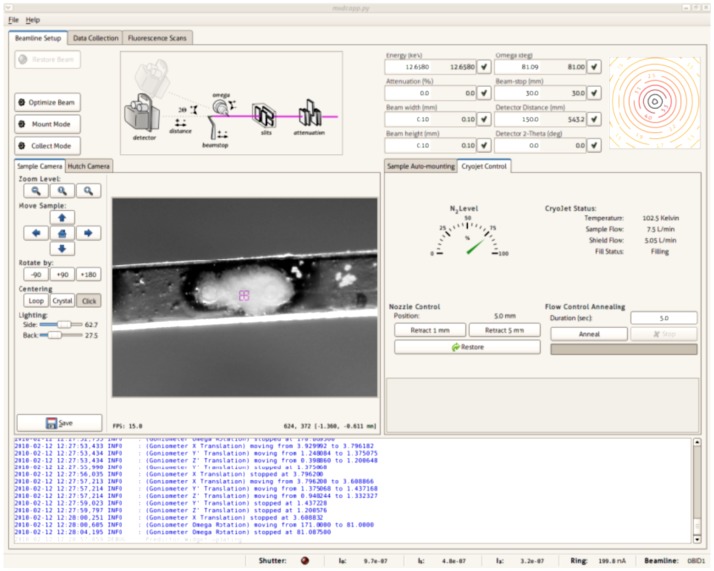
The beamline setup, sample camera and cryoject control tabs of the MxDC software at the 08ID-1 beamline. A view from sample camera shows two oocytes on cryotop and square in the middle shows the path and size (0.1×0.1 mm) of X-ray beam.

SXRD was performed at Canadian Macromolecular Crystallography Facility (CMCF), Canadian Light Source (http://www.lightsource.ca) located on the University of Saskatchewan campus in Saskatoon, using a fully automated synchrotron beamline 08ID-1. The details of beamline 08ID-1 and CMCF have been reported earlier [37], [38]. Briefly, Canadian Light Source is a 2.9 GeV national synchrotron radiation facility. The beamline 08ID-1 is illuminated by a hybrid small-gap in-vacuum undulator. The end-station of beamline 08ID-1 is equipped with a Rayonix MX300 HD CCD X-ray detector; a Huber 410 single axis goniometer; on-axis sample visualization system and CryoJet (Oxford Instruments) (for details, see Fig. 2). The typical size of beamline 08ID-1 is 0.1 mm with a passing flux of 2×10^12^ photons/s. The CMCF software developed in-house has three fully integrated components, i.e. 1) MX Data Collector (MxDC), a beamline control system integrated with data processing module; 2) AutoProcess, a fully automated data collection and processing system with minimum human intervention; and 3) Sample management and remote monitoring system (**Fig. 3**) [39].

### Synchrotron X-ray diffraction of water, TCM-199 and VS2 for detection of glass or ice formation

This experiment was conducted as a proof of ice formation in water and TCM-199 and glass formation in VS2. The cryoloop was immersed in deionized water, TCM-199 or VS2 solutions (22°C), directly plunged in liquid nitrogen and quickly mounted on the goniometer head with an ultra-cooled CryoTong (Hampton Research) (**S1 Figure**).

### Synchrotron X-ray diffraction of bovine COCs and morulae for detection of glass or ice formation

The cryovial, containing cryotop with bovine COCs or morulae, were removed from liquid nitrogen storage tank into a wide mouth thermos flask. CrystalCap Copper Magnetic was dislodged from cryovial with an ultra-cooled CrystalWand Magnetic (Hampton Research) and quickly mounted on the goniometer head with an ultra-cooled CryoTong (Hampton Research) (**S1 Figure**).

After mounting CrystalCap, cryoloop or cryotop propylene strip was aligned to X-ray beam using on-axis sample visualization system. The sample was positioned in the center of X-ray path by moving and rotating the goniometer head using the remote monitoring system (**S1 Video**). SXRD was conducted using following beamline set up: energy 12.6580 keV, pixel size of the X-ray CCD detector 0.073242 mm, beam stop's distance from sample 30 mm, beam size 0.1×0.1 mm, angle of incidence 90°, sample to detector distance 150 mm, exposure time 1 sec. The sample temperature was maintained at 102 K (−171°C) with a constant flow of temperature-stabilised liquid nitrogen vapors (7.5 L/min) using CryoJet Controller (Oxford Instruments, Austin, TX).

Data were collected with in-house developed MxDC, a component of CMCF software system. The 2D data were analyzed for Debye's rings using Diffraction Image Viewer software developed at Canadian Macromolecular Crystallography Facility, Canadian Light Source. The 2D data from TCM alone and the non-vitrified (control) COCs were submitted to International Center for Diffraction Data (ICDD) for comparison with the published data on powder diffraction. The 2D data were converted to 1-dimensional (1D) d-spacing (Å) scans using FIT2D software developed at European Synchrotron Radiation Facility (ESRF) [40]. **S1 Figure** and **S1 Video** are available online at www.plosone.org.

### Statistical analysis

SXRD analysis of water, TCM-199 and VS2 was repeated twice on different batches of solutions. Five COCs and morulae (vitrified and non-vitrified each) were studied for SXRD analysis, on different dates (replicates). The intensities of ice peaks in the non-vitrified control and vitrified COCs were compared with student t-test using SYSTAT statistical software (SPSS, Chicago, IL).

## Results

### Synchrotron X-ray diffraction of water, TCM-199 and VS2 for detection of glass or ice formation

**Figure 4:**
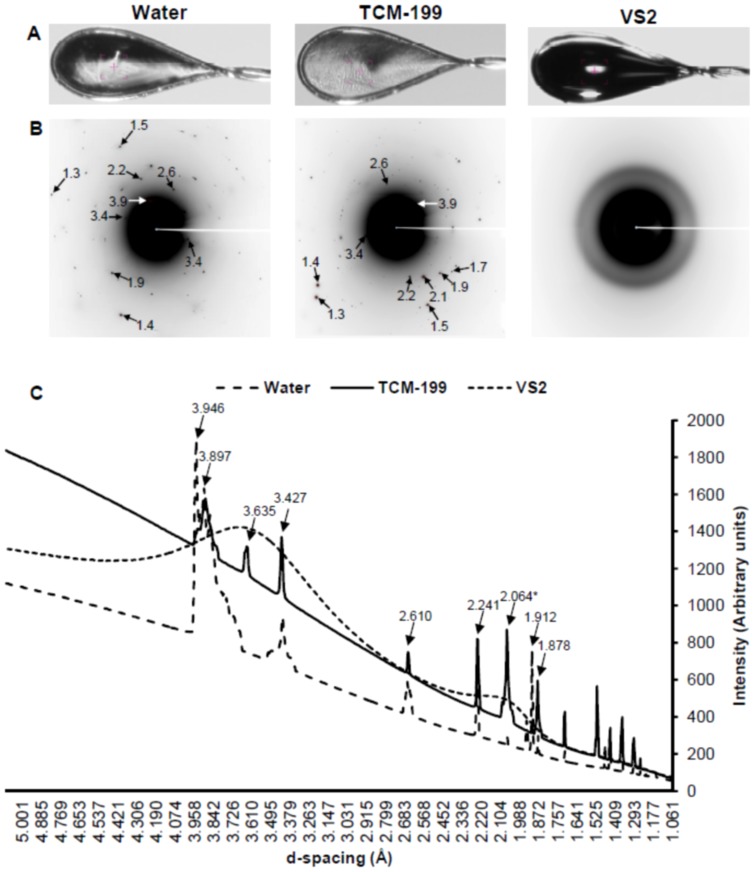
Physical appearance, 2D SXRD and 1D SXRD of Water, TCM-199 and VS2 in cryoloops at 102K. A) Appearnce of solutions through sample camera. B) Representative 2D SXRD with display of d-spacing (Å). C) Representative 1D SXRD indicating the relative intensities of peaks vs. d-spacing (Å). As d-spacing increases, the diameter of Debye's ring decreases. All peaks in water and TCM-199 corresponded to the hexagonal ice crystals except one peak (*) in TCM-199 which corresponded to NaH_2_PO_4_.H_2_O.

Water, TCM-199 and VS2 represented exactly similar Debye's rings and ice peaks between replicates; therefore, their representative physical appearance, 2D and 1D SXRD are shown in **Fig. 4**. The physical appearance of water and TCM showed ice crystal formation at 102K. The 2D SXRD data revealed Debye's rings, corresponding to the hexagonal ice crystals, at 3.897, 3.635, 3.427, 2.610, 2.241, 1.912 and 1.878 Å in both water and TCM-199. The ice peak at 3.946 Å was present in water only. Data from ICDD confirmed all hexagonal ice peaks and an additional peak at 2.064 Å corresponding to sodium phosphate monobasic hydrate (NaH_2_PO_4_.H_2_O) in TCM-199. In contrast, VS2 showed typical glass appearance at 102K (**Fig. 4A**). The 2D and 1D SXRD data of VS2 did not show any Debye's ring and ice peaks rather exhibited smooth curves representing typical amorphous phase (**Fig. 4B and 4C**).

### Synchrotron X-ray diffraction of bovine COCs and morulae for detection of glass or ice formation

**Figure 5:**
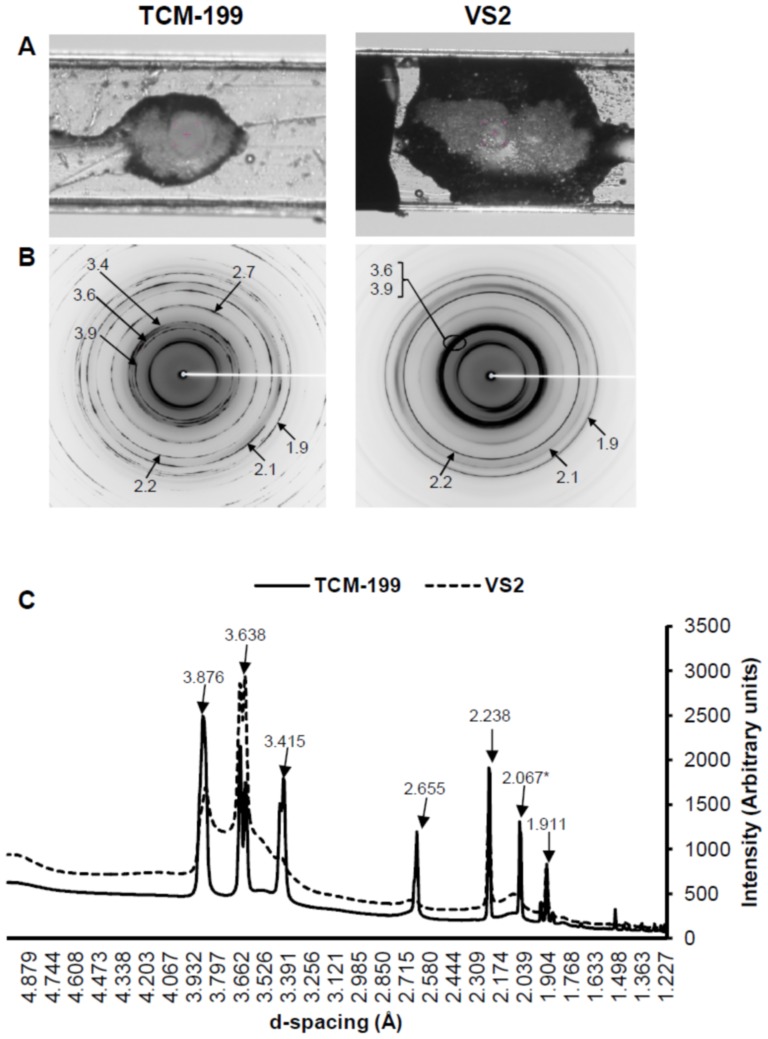
Physical appearance, 2D SXRD and 1D SXRD of bovine COCs in TCM-199 (non-vitrified control) and VS2 (vitrified) on cryotops at 102K. A) Appearance of COCs through sample camera. B) Representative 2D SXRD with display of d-spacing. C) Representative 1D SXRD indicating the relative intensities of peaks vs. d-spacing. All peaks corresponded to the hexagonal ice crystals except one peak (*) which corresponded to NaH_2_PO_4_.H_2_O.

**Figure 6:**
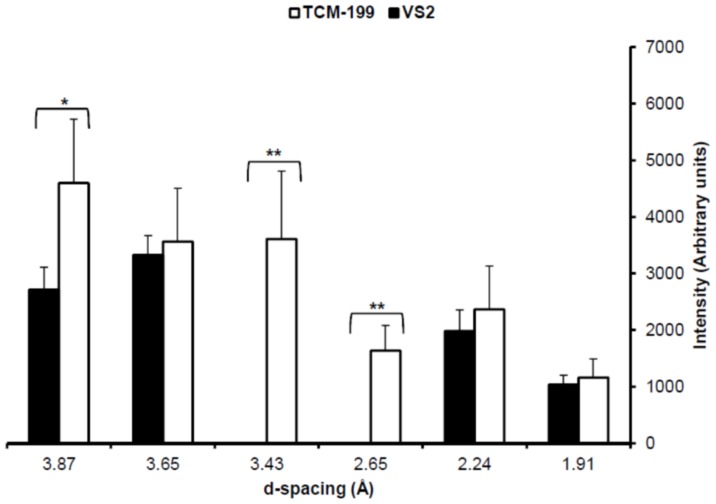
Ice peaks' intensities in bovine COCs in TCM-199 (non-vitrified control) or VS2 (vitrified). Each bar represents mean±SEM (N = 5 COCs in each group). Asterisk (*) denotes the significant difference (P<0.05) in ice peak intensity between non-vitrified and vitrified COCs at a given d-spacing. Double asterisks (**) denote the presence of ice peaks in the non-vitrified COCs only.

The non-vitrified COCs in TCM-199 clearly showed normal size with icy appearance whereas the vitrified COCs in VS2 reduced in size and appeared glassy (**Fig. 5A**). SXRD data demonstrated the common ice peaks between the non-vitrified and vitrified COCs at 3.876, 3.638, 2.238 and 1.911 Å (**Fig. 5B and 5C**). The intensities of ice peaks did not differ statistically between the non-vitrified and vitrified COCs except an ice peak at 3.876 Å was higher (P<0.05) in the non-vitrified than vitrified COCs (**Fig. 6**). Two ice peaks at 3.415 and 2.655 Å were present in the non-vitrified COCs only (**Fig. 5C** **and** **6**). The additional peak (2.067 Å) corresponding to NaH_2_PO_4_.H_2_O, found in TCM-199, was also present in the non-vitrified COCs. All Debye's rings and peaks corresponding to the hexagonal ice and NaH_2_PO_4_.H_2_O in the non-vitrified control COCs were also confirmed by ICDD.

**Figure 7:**
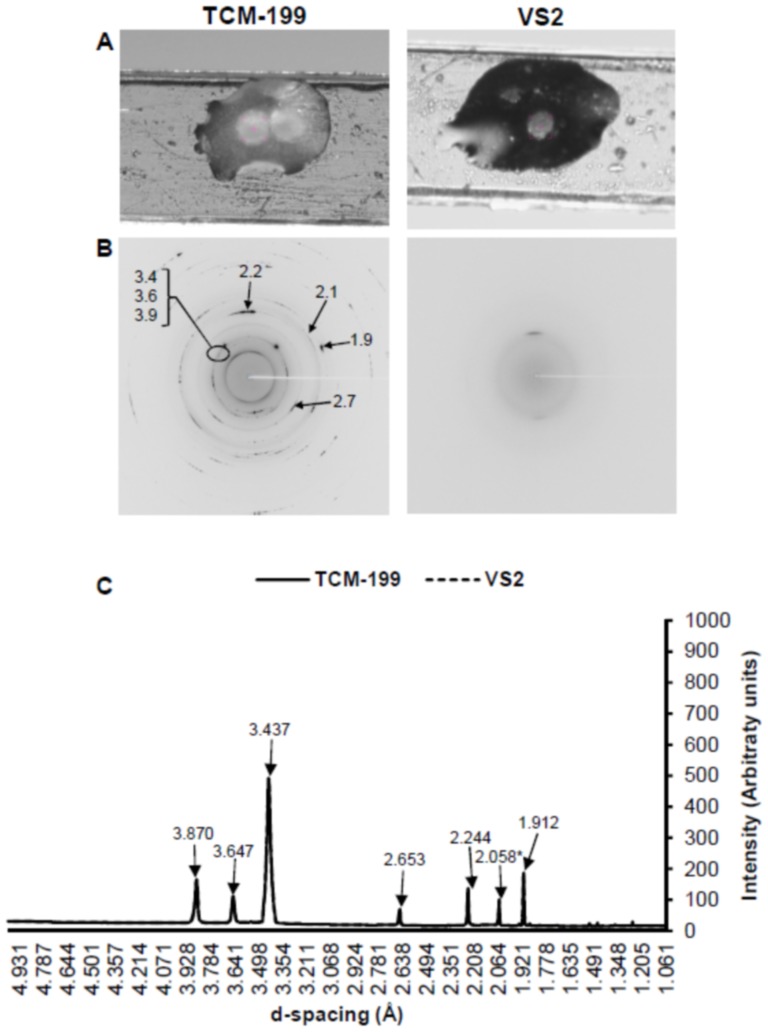
Physical appearance, 2D SXRD and 1D SXRD of bovine morulae in TCM-199 (non-vitrified control) and VS2 (vitrified) on cryotops at 102K. A) Appearance of morula through sample camera. B) Representative 2D SXRD with display of d-spacing (Å). C) Representative 1D SXRD indicating the relative intensities of peaks vs. d-spacing (Å). All peaks corresponded to the hexagonal ice crystals except one peak (*) which corresponded to NaH_2_PO_4_.H_2_O.

Like COCs, the non-vitrified bovine morulae in TCM-199 showed normal size and extracellular ice crystal formation (**Fig. 7A**). The Debye's rings and peaks corresponding to the hexagonal ice crystals (3.870, 3.647, 3.437, 2.653, 2.244 and 1.912 Å) and NaH_2_PO_4_.H_2_O (2.058 Å) were present in the non-vitrified morulae (**Fig. 7B and 7C**). Bovine morulae in VS2 reduced in size and appeared glassy, and there was no detectable Debye's ring and ice peaks in the vitrified morulae.

## Discussion

This is the first study on the determination of vitrifiability of vitrification solution, and the confirmation of glass or ice formation in the vitrified bovine COCs and early embryos (morulae) at ultra-low temperature (102 K) using synchrotron X-ray diffraction (SXRD) method. SXRD confirmed the vitrifiability of VS2 used in this study for vitrification of bovine COCs and morulae. The non-vitrified COCs in TCM-199 exhibited Debye's ring and peaks corresponding to the hexagonal ice crystals and NaH_2_PO_4_.H_2_O. The vitrified COCs in VS2 demonstrated randomly distributed fine ice crystals and thus did not undergo true vitrification. In contrast, the vitrified bovine morulae underwent typical vitrification.

This study provided an evidence of the ice crystal formation in water and TCM-199, and the glass formation in a commonly used VS2 for bovine COCs and embryos. The “grainy” structures in Debye's rings in water and TCM-199 indicated their polycrystalline nature with a limited number of individual crystals. Therefore, 1D SXRD data for a “grainy” 2D data was only useful for peak identification, but not for evaluation of relative intensity of peaks. In this study, the majority of Debye's rings, representing the hexagonal ice crystals, were common between water and TCM-199. Similar ice peaks, with slight differences in d-spacing by a fraction of Å, have been reported earlier in water at temperature 98 K [41]. The addition of permeating cryoprotectants increases the viscosity of solution which turns into glass upon ultra-rapid cooling. Normally, the vitrifiability, glass stability and toxicity of a vitrification solution are predicted based on the mathematical calculations for water permeability, solute permeability and activation energy [42]. SXRD data showed the amorphous behaviour of VS2 upon vitrification and did not demonstrate any sharp ice peak, as found in water and TCM-199. In a typical diffraction study, the crystalline and amorphous materials are characterized by sharp peaks and smooth curve-like pattern respectively [30]. Visual appearance of the non-vitrified and vitrified solutions in liquid nitrogen with naked eye has been reported earlier [7], [17], [43]. During conventional freezing, the growth of intracellular ice crystal formation has been observed under cryomicroscope [44], [45]. However, both methods did not yield the detailed information on ice peaks in cryoprotectant solutions and tissues.

In this study, bovine COCs (GV stage) were selected due to their easy accessibility and vitrification in the field conditions. The mature oocyte (MII stage) could be another alternate to preserve the female genetics but meiotic spindle at this stage is more sensitive to chilling [46], [47]. Although the vitrified COCs shrunk in response to osmotic gradient across membrane but they exhibited similar ice peaks in the non-vitrified control COCs except two peaks at 3.43 and 2.65 Å were missing. An intracellular origin of these ice crystals in so-called vitrified COCs is anticipated because extracellular VS2 turned into glass, as demonstrated in the first experiment. Furthermore, it is expected that these ice crystals, formed inside the cumulus cells and/or oocyte, damage the organelles leading to poor oocytes' maturation, cleavage and embryo development. The ice formation in COCs suggested the permeating cryoprotectants in VS2, in spite of possessing vitrifiability, could not necessarily penetrate throughout COCs and the critical concentration of cryoprotectants required for intracellular vitrification, could not be achieved. It could be due to the slow permeability of plasma membrane of mammalian oocytes [9]. It should be kept in mind that COCs have large number of surrounding cumulus cells which retard the penetration of permeating cryoprotectants [4], [48]. In this study, the vitrified bovine morulae served as an internal control since morulae survive vitrification better than oocytes [1], [5], [6], [49]. Morulae not only shrunk but also avoided the hexagonal ice crystal formation and underwent typical vitrification. Our data also suggested the success of vitrification depends upon type of cells/tissues to be vitrified.

In addition to several ice peaks, a peak corresponding to NaH_2_PO_4_.H_2_O was also observed in TCM-199, non-vitrified COCs and non-vitrified morulae. TCM-199 is a common base-medium for bovine oocyte maturation, fertilization, embryo culture, and for preparation of vitrification solutions. The bulk portion of TCM-199 is composed of water which demonstrated several Debye's rings and ice peaks. Sodium phosphate monobasic is one of the inorganic salts present in TCM-199 along with amino acids, vitamins and other components. The Debye's rings and peaks associated with the hexagonal ice crystals and NaH_2_PO_4_.H_2_O in TCM-199 and the non-vitrified control COCs were confirmed by ICDD. The Debye's rings at the lower end of d-spacing (<1.9 Å) did not match with any powder diffraction files.

The potential side effect of X-ray diffraction is an intracellular damage leading to malfunctioning of cells, tissues or organisms after their exposure to X-ray [50]. However, cryocooling of a sample to 100K or below reduces the radiation damage to cells or tissues [51]. In future, it will be interesting to study the maturation and fertilization abilities of COCs and developmental competence of early embryos following exposure to synchrotron X-ray.

## Conclusions

SXRD is useful to determine the vitrifiability of any solution and its efficiency in vitrifying various cells/tissues. SXRD can be used to detect glass or ice formation in the vitrified and non-vitrified cells/tissues, respectively. This study indicated that bovine COCs at GV stage did not transform into glass upon vitrification rather produced randomly distributed fine ice crystals; whereas, bovine morulae turned into a glass. The strenuous attempts should be made to improve the vitrification of bovine COCs by developing a suitable cryoprotectant solution, and optimum cooling and warming rates.

## Supporting Information

S1 Figure **Handling of samples in liquid nitrogen.** CrystalWand Magnetic holding CrystalCap Magnetic, containing either cryoloop or cryotop, in a thermos flask filled with liquid nitrogen. After fixing CryoTong around CrystalCap, the CrsytalWand is dislodged and CrystalCap is quickly transferred to the goniometer head on the end station of O8ID-1beamline.
(TIF)

S1 Video **Remote monitoring and rotation of vitrified COCs on the modified cryotop.** This procedure helped to visualize and position the sample in the center of X-ray path.
(MPG)

## References

[1] Pereira RM, Marques CC (2008) Animal oocyte and embryo cryopreservation. Cell Tissue Banking 9:267–277. 18496769 10.1007/s10561-008-9075-2

[2] Prentice JR, Anzar M (2010) Cryopreservation of mammalian oocyte for conservation of animal genetics. Vet Med Int 2011. 10.4061/2011/146405 PMC2945659 20886016

[3] Hunter JE, Fuller BJ, Bernard A, Jackson A, Shaw RW (1995) Vitrification of human oocytes following minimal exposure to cryoprotectants; initial studies on fertilization and embryonic development. Hum Reprod 10:1184–1188. 7657762 10.1093/oxfordjournals.humrep.a136115

[4] Albarracin JL, Morato R, Izquierdo D, Mogas T (2005) Vitrification of calf oocytes: effects of maturation stage and prematuration treatment on the nuclear cytoskeletal components of oocytes and their subsequent development. Mol Reprod Dev 72:239–249. 15968627 10.1002/mrd.20326

[5] Martino A, Songsasen N, Leibo SP (1996) Development into blastocysts of bovine oocytes cryopreserved by ultra-rapid cooling. Biol Reprod 54:1059–1069. 8722627 10.1095/biolreprod54.5.1059

[6] Men H, Monson RL, Rutledge JJ (2002) Effect of meiotic stages and maturation protocols on bovine oocyte's resistance to cryopreservation. Theriogenology 57:1095–1103. 12041903 10.1016/s0093-691x(01)00679-3

[7] Liebermann J, Dietl J, Vanderzwalmen P, Tucker MJ (2003) Recent developments in human oocyte, embryo and blastocyst vitrification: where are we now? Reprod Biomed Online 7:623–633. 14748959 10.1016/s1472-6483(10)62084-6

[8] Massip A (2003) Cryopreservation of bovine oocytes: current status and recent developments. Reprod Nutr Dev 43:325–330. 14971824 10.1051/rnd:2003024

[9] Saragusty J, Arav A (2011) Current progress in oocyte and embryo cryopreservation by slow freezing and vitrification. Reproduction 141:1–19. 20974741 10.1530/REP-10-0236

[10] Kasai M, Ito K, Edashige K (2002) Morphological appearance of the cryopreserved mouse blastocyst as a tool to identify the type of cryoinjury. Hum Reprod 17:1863–1874. 12093853 10.1093/humrep/17.7.1863

[11] Mazur P (1970) Cryobiology: the freezing of biological systems. Science 168:939–949. 5462399 10.1126/science.168.3934.939

[12] Rall WF, Fahy GM (1985) Ice-free cryopreservation of mouse embryos at -196°C by vitrification. Nature 313:573–575. 3969158 10.1038/313573a0

[13] Arav A, Shehu D, Mattioli M (1993) Osmotic and cytotoxic study of vitrification of immature bovine oocytes. J Reprod Fertil 99:353–358. 8107016 10.1530/jrf.0.0990353

[14] Vajta G, Holm P, Kuwayama M, Booth PJ, Jacobsen H, et al (1998) Open Pulled Straw (OPS) vitrification: a new way to reduce cryoinjuries of bovine ova and embryos. Mol Reprod Dev 51:53–58. 9712317 10.1002/(SICI)1098-2795(199809)51:1<53::AID-MRD6>3.0.CO;2-V

[15] Kuwayama M (2007) Highly efficient vitrification for cryopreservation of human oocytes and embryos: the cryotop method. Theriogenology 67:73–80. 17055564 10.1016/j.theriogenology.2006.09.014

[16] Zhou XL, Al Naib A, Sun DW, Lonergan P (2010) Bovine oocyte vitrification using the Cryotop method: effect of cumulus cells and vitrification protocol on survival and subsequent development. Cryobiology 61:66–72. 20510225 10.1016/j.cryobiol.2010.05.002

[17] Larman M, Gardner D (2014) Ultrarapid vitrification of mouse oocytes and embryos. In: Lewandoski M, editor. Mouse molecular embryology.Springer US. pp.153–165. 10.1007/978-1-60327-292-6_10 24318819

[18] Nakagata N (1989) High survival rate of unfertilized mouse oocytes after vitrification. J Reprod Fertil 87:479–483. 2600904 10.1530/jrf.0.0870479

[19] Fuku E, Kojima T, Shioya Y, Marcus GJ, Downey BR (1992) *In vitro* fertilization and development of frozen-thawed bovine oocytes. Cryobiology 29:485–492. 1395686 10.1016/0011-2240(92)90051-3

[20] Prentice JR, Singh J, Dochi O, Anzar M (2011) Factors affecting nuclear maturation, cleavage and embryo development of vitrified bovine cumulus-oocyte complexes. Theriogenology 75:602–609. 21190729 10.1016/j.theriogenology.2010.09.027

[21] Prentice-Biensch JR, Singh J, Mapletoft RJ, Anzar M (2012) Vitrification of immature bovine cumulus-oocyte complexes: effects of cryoprotectants, the vitrification procedure and warming time on cleavage and embryo development. Reprod Biol Endocrinol 10:73. 22954348 10.1186/1477-7827-10-73 PMC3814649

[22] Diez C, Duque P, Gomez E, Hidalgo CO, Tamargo C, et al (2005) Bovine oocyte vitrification before or after meiotic arrest: effects on ultrastructure and developmental ability. Theriogenology 64:317–333. 15955356 10.1016/j.theriogenology.2004.11.023

[23] Morato R, Mogas T, Maddox-Hyttel P (2008) Ultrastructure of bovine oocytes exposed to taxol prior to OPS vitrification. Mol Reprod Dev 75:1318–1326. 18247367 10.1002/mrd.20873

[24] Hyttel P, Vajta G, Callesen H (2000) Vitrification of bovine oocytes with the open pulled straw method: ultrastructural consequences. Mol Reprod Dev 56:80–88. 10737970 10.1002/(SICI)1098-2795(200005)56:1<80::AID-MRD10>3.0.CO;2-U

[25] Leibo SP (2008) Cryopreservation of oocytes and embryos: optimization by theoretical versus empirical analysis. Theriogenology 69:37–47. 18023472 10.1016/j.theriogenology.2007.10.006

[26] Arav A (2014) Cryopreservation of oocytes and embryos. Theriogenology 81:96–102. 24274414 10.1016/j.theriogenology.2013.09.011

[27] Seki S, Mazur P (2012) Ultra-rapid warming yields high survival of mouse oocytes cooled to -196°C in dilutions of a standard vitrification solution. PLOS ONE 7:e36058. 22558325 10.1371/journal.pone.0036058 PMC3338624

[28] Arav A, Saragusty J (2013) Directional freezing of spermatozoa and embryos. Reprod Fertil Dev 26:83–90. 24305180 10.1071/RD13295

[29] Will G (2006) Powder Diffraction: the Rietveld method and the two stage method to determine and refine crystal structures from powder diffraction data. New York: Springer Science+Business Media LLC. 234 p.

[30] Nunes C, Mahendrasingam A, Suryanarayanan R (2005) Quantification of crystallinity in substantially amorphous materials by synchrotron X-ray powder diffractometry. Pharm Res 22:1942–1953. 16132342 10.1007/s11095-005-7626-9

[31] Varshney DB, Elliott JA, Gatlin LA, Kumar S, Suryanarayanan R, et al (2009) Synchrotron X-ray diffraction investigation of the anomalous behavior of ice during freezing of aqueous systems. J Phys Chem B 113:6177–6182. 19358549 10.1021/jp900404m

[32] Varshney DB, Sundaramurthi P, Kumar S, Shalaev EY, Kang SW, et al (2009) Phase transitions in frozen systems and during freeze-drying: quantification using synchrotron X-ray diffractometry. Pharm Res 26:1596–1606. 19326191 10.1007/s11095-009-9868-4

[33] Varshney D, Kumar S, Shalaev E, Kang S-W, Gatlin L, et al (2006) Solute crystallization in frozen systems–use of synchrotron radiation to improve sensitivity. Pharm Res 23:2368–2374. 16927181 10.1007/s11095-006-9051-0

[34] Parrish JJ, Krogenaes A, Susko-Parrish JL (1995) Effect of bovine sperm separation by either swim-up or Percoll method on success of *in vitro* fertilization and early embryonic development. Theriogenology 44:859–869. 16727781 10.1016/0093-691x(95)00271-9

[35] Brackett BG, Oliphant G (1975) Capacitation of rabbit spermatozoa *in vitro* . Biol Reprod 12:260–274. 1122333 10.1095/biolreprod12.2.260

[36] Chian R, Kuwayama M, Tan L, Tan J, Kato O, et al (2004) High survival rate of bovine oocytes matured *in vitro* following vitrification. J Reprod Dev 50:685–696. 15647621 10.1262/jrd.50.685

[37] Grochulski P, Fodje M, Labiuk S, Gorin J, Janzen K, et al (2012) Canadian macromolecular crystallography facility: a suite of fully automated beamlines. J Struct Funct Genomics 13:49–55. 22270456 10.1007/s10969-012-9123-9

[38] Grochulski P, Fodje MN, Gorin J, Labiuk SL, Berg R (2011) Beamline 08ID-1, the prime beamline of the Canadian Macromolecular Crystallography Facility. J Synchrotron Radiat 18:681–684. 21685687 10.1107/S0909049511019431

[39] Fodje M, Janzen K, Berg R, Black G, Labiuk S, et al (2012) MxDC and MxLIVE: software for data acquisition, information management and remote access to macromolecular crystallography beamlines. J Synchrotron Radiat 19:274–280. 22338690 10.1107/S0909049511056305

[40] Hammersley AP, Svensson SO, Hanfland M, Fitch AN, Hausermann D (1996) Two-dimensional detector software: from real detector to idealised image or two-theta scan. High Pressure Res 14:235–248.

[41] Dowell LG, Rinfret AP (1960) Low-temperature forms of ice as studied by X-ray diffraction. Nature 188:1144–1148.

[42] Yavin S, Arav A (2007) Measurement of essential physical properties of vitrification solutions. Theriogenology 67:81–89. 17070573 10.1016/j.theriogenology.2006.09.029

[43] Weiss ADH, Fraser Forbes J, Scheuerman A, Law GK, Elliott JAW, et al (2010) Statistical prediction of the vitrifiability and glass stability of multi-component cryoprotective agent solutions. Cryobiology 61:123–127. 20558152 10.1016/j.cryobiol.2010.05.008

[44] Leibo SP, McGrath JJ, Cravalho EG (1975) Microscopic observation of intracellular ice formation in mouse ova as a function of cooling rate. Cryobiology 12:579. 10.1016/0011-2240(78)90036-6 710156

[45] Yang C-Y, Yeh Y-HF, Lee P-T, Lin T-T (2013) Effect of cooling rate and cryoprotectant concentration on intracellular ice formation of small abalone (*Haliotis diversicolor*) eggs. Cryobiology 67:7–16. 23619025 10.1016/j.cryobiol.2013.04.003

[46] Wu B, Tong J, Leibo SP (1999) Effects of cooling germinal vesicle-stage bovine oocytes on meiotic spindle formation following *in vitro* maturation. Mol Reprod Dev 54:388–395. 10542379 10.1002/(SICI)1098-2795(199912)54:4<388::AID-MRD9>3.0.CO;2-7

[47] Abe Y, Hara K, Matsumoto H, Kobayashi J, Sasada H, et al (2005) Feasability of a nylon mesh holder for vitrification of bovine germinal vesicle oocytes in subsequent production of viable blastocysts. Biol Reprod 72:1416–1420. 15689537 10.1095/biolreprod.104.037051

[48] Minasi MG, Fabozzi G, Casciani V, Ferrero S, Litwicka K, et al (2012) Efficiency of slush nitrogen vitrification of human oocytes vitrified with or without cumulus cells in relation to survival rate and meiotic spindle competence. Fertil Steril 97:1220–1225. 22401811 10.1016/j.fertnstert.2012.02.022

[49] Shaw JM, Oranratnachai A, Trounson AO (2000) Fundamental cryobiology of mammalian oocytes and ovarian tissue. Theriogenology 53:59–72. 10735062 10.1016/s0093-691x(99)00240-x

[50] Kasai N, Kakudo M (2010) X-Ray diffraction by macromolecules. Berlin-Heidelberg: Springer. 504 p.

[51] Snell EH, Bellamy HD, Rosenbaum G, van der Woerd MJ (2007) Non-invasive measurement of X-ray beam heating on a surrogate crystal sample. J Synchrotron Radiat 14:109–115. 17211077 10.1107/S090904950604605X

