# Supplementary figures and images for: Synchrotron X-Ray Diffraction to Detect Glass or Ice Formation in the Vitrified Bovine Cumulus-Oocyte Complexes and Morulae
Source: PLoS One. 2014 Dec 23;9(12):e114801. doi: 10.1371/journal.pone.0114801 (PMC4275205; doi:10.1371/journal.pone.0114801)

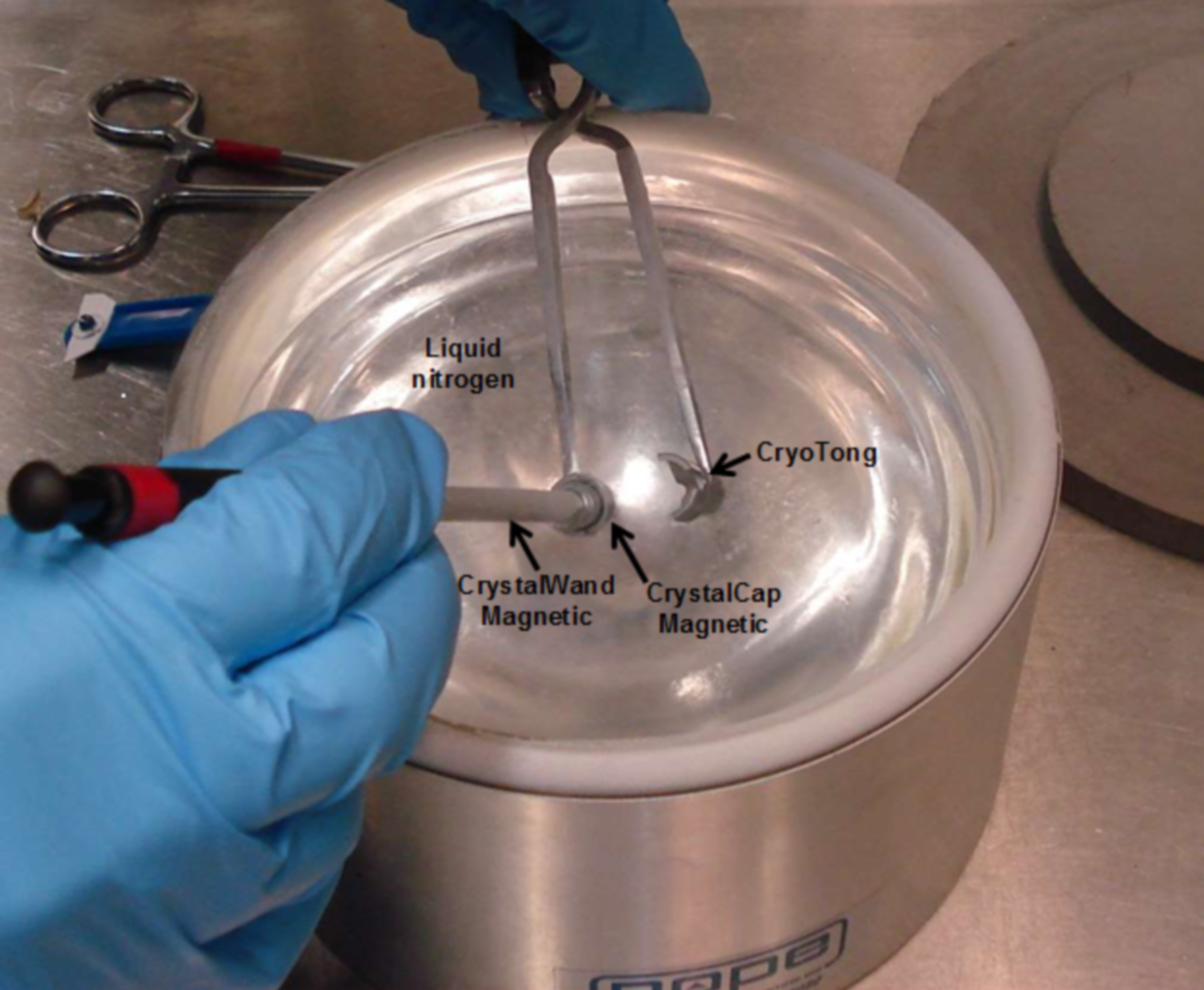

Supplement: S1 Figure — Handling of samples in liquid nitrogen. CrystalWand Magnetic holding CrystalCap Magnetic, containing either cryoloop or cryotop, in a thermos flask filled with liquid nitrogen. After fixing CryoTong around CrystalCap, the CrsytalWand is dislodged and CrystalCap is quickly transferred to the goniometer head on the end station of O8ID-1beamline. (TIF) [file pone.0114801.s001.tif]
